# A Review of Mechanical and Chemical Sensors for Automotive Li-Ion Battery Systems

**DOI:** 10.3390/s22051763

**Published:** 2022-02-24

**Authors:** Matteo Dotoli, Riccardo Rocca, Mattia Giuliano, Giovanna Nicol, Flavio Parussa, Marcello Baricco, Anna Maria Ferrari, Carlo Nervi, Mauro Francesco Sgroi

**Affiliations:** 1 Centro Ricerche FIAT S.C.p.A., 10043 Orbassano, Italy; mattia.giuliano@crf.it (M.G.); giovanna.nicol@crf.it (G.N.); flavio.parussa@crf.it (F.P.); mauro.sgroi@crf.it (M.F.S.); 2 Department of Chemistry and NIS—INSTM, University of Turin, 10125 Torino, Italy; marcello.baricco@unito.it (M.B.); anna.ferrari@unito.it (A.M.F.); carlo.nervi@unito.it (C.N.)

**Keywords:** Li-ion battery system, safety, state of health, chemical sensors

## Abstract

The electrification of passenger cars is one of the most effective approaches to reduce noxious emissions in urban areas and, if the electricity is produced using renewable sources, to mitigate the global warming. This profound change of paradigm in the transport sector requires the use of Li-ion battery packages as energy storage systems to substitute conventional fossil fuels. An automotive battery package is a complex system that has to respect several constraints: high energy and power densities, long calendar and cycle lives, electrical and thermal safety, crash-worthiness, and recyclability. To comply with all these requirements, battery systems integrate a battery management system (BMS) connected to an complex network of electric and thermal sensors. On the other hand, since Li-ion cells can suffer from degradation phenomena with consequent generation of gaseous emissions or determine dimensional changes of the cell packaging, chemical and mechanical sensors should be integrated in modern automotive battery packages to guarantee the safe operation of the system. Mechanical and chemical sensors for automotive batteries require further developments to reach the requested robustness and reliability; in this review, an overview of the current state of art on such sensors will be proposed.

## 1. Introduction

**Figure 1:**
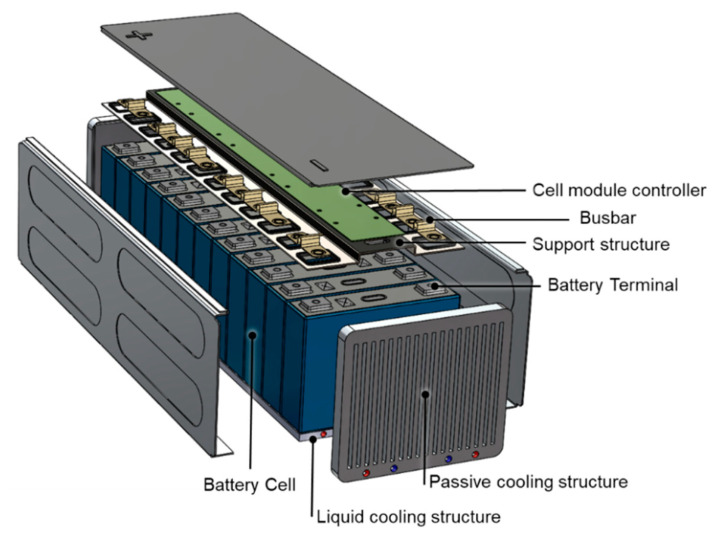
Typical layout of an automotive battery package integrating prismatic-type lithium ion cells. The thermal management system is also shown. Figure partially reproduced from [8].

One of the most common strategies to mitigate global warming and to reduce the noxious emissions in urban areas is the electrification of passenger and industrial vehicles. This requires the production of energy storage systems, based on Li-ion cells, characterized by high energy and power densities, long cycle life, and high safety. The integration of such battery packages on a vehicle imposes strict control on its safety status, state of charge (SoC), and state of the health (SoH) during the operative life of the vehicle. The state of health is defined as the ratio between the actual and initial capacity of the battery and is an indicator of the expected residual life of the system. Due to the cost limitations and to the need of reducing the complexity, the unequivocal and robust determination of SoC and SoH of the automotive battery package is a difficult task.The interested reader can find more details in [1,2]. Moreover, fast charge of the energy storage system is one of main customer expectations: this requires one to protect the Li-ion cells from the formation of Li dendrites at the anode and from temperature peaks [3]. Finally, an automotive battery package has to be resistant to crash events, has to guarantee electric insulation, and has to integrate insulating materials to be able to slow down a possible thermal runaway event. All these requirements can be satisfied only by integrating in the system a complex network of sensors and a heat conditioning system controlled by a battery management system (BMS). The reliability of BMS and related software has increased in the last decade due to the intense research efforts of producers and academic groups [4,5,6]. Figure 1 shows a typical automotive battery package integrating Li-ion cells with rigid prismatic packaging. We have recently briefly summarized the main needs arising from the automotive applications and reviewed the most important and promising technological solutions [7].

**Figure 2:**
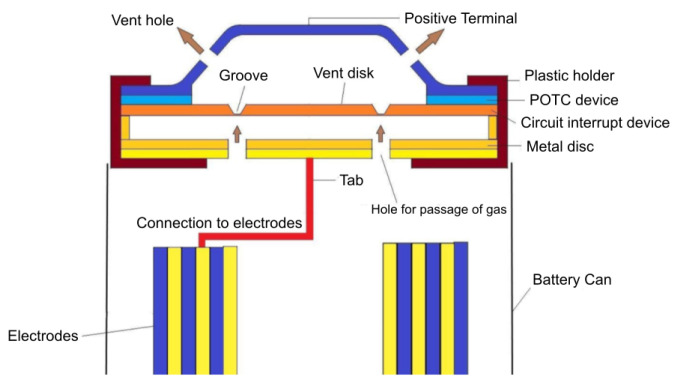
Schematic structure of Circuit Interrupt Device (CID), Positive Temperature Coefficient (PTC), and venting system in a cylindrical cell. Reproduced from [13].

The state-of-the-art technologies implemented at a cell level to increase the safety of battery systems are represented by passive safety devices aiming at preventing thermal runaway events. Passive Current Interrupt Devices (CID) are convenient and efficient protection devices, usually integrated in commercial cylindrical Li-ion cells [9]. The CID mainly consist of four components: a conductive flexible membrane as a top disk, a second plastic insert, a bottom disk, and a metallic foil. The metallic foil is welded between the top and bottom disk through the central opening of the bottom disk. The plastic insert has the purpose to electrically insulate the remaining area between the top and bottom disk. The welded central point of the system is the weak point; it is electrically connected to the tab and electrochemical system. Whenever an abusive condition occurs, for example, an overcharging, the large amount of gases produced generates an over-pressure. If the internal pressure rises up to 1.0–1.2 MPa, the top disk moves upwards, breaking the weak point. Suddenly, the electrical circuit is interrupted; no electricity can pass through the system, and the electrochemical reactions inside the cell are arrested. Such systems are widely implemented in cylindrical cells, but several applications in prismatic cells are also reported [10]. Another useful passive safety element present inside commercial cells is commonly known as PTC (Positive Temperature Coefficient) device [11]. It is usually included into the top cap, between the positive terminal and the CID burst disk. It has a ring shape, and it is composed of a polymeric material with conductive additives (carbon black) dispersed inside the polymeric matrix (polypropylene or low-density polyethylene) [12]. The PTC has the function to limit the current flow in case of overload or accidental short circuit, thus avoiding overheating. In contrast to CID, PTC works in a reversible way by changing its electrical resistance with the temperature. Beyond a limit current value, the PTC resistance increases by several magnitude because of the Joule heating effect and limits the current. After the reduction of current/temperature, the resistance of PTC drops again. Figure 2 shows the cross-section of a cylindrical cell integrating PTC and CID devices.

Clearly, passive devices are not sufficient to guarantee the safety and the control state of health and state of charge of the complex automotive battery systems. On-board electrical [14,15,16] and thermal measurements [17,18] are based on well established technologies that can be effectively integrated in automotive battery packages. The main physical quantities used to characterize and simulate the battery behavior are the current, the electric potential, and the temperature. The electric potential is commonly measured at the cell level using appropriate integrated circuits [19]. On the other hand, the measurement of the current is more complex and usually realized using high-precision shunt resistors or Hall-effect sensors. The first, even if they dissipate the energy stored in the cell, are characterized by simplicity, low cost, and good accuracy (up to 0.1% or better) [20]. On the other hand, induction Hall-effect sensors, lacking any electrical connection with the cells, do not interfere with the circuitry of the battery system; their main drawbacks are related to the limited accuracy and to the interference of external magnetic fields [21,22]. The reliability and fault statistics of current sensors for battery applications were recently discussed in great detail by Hu et al. [23]. The accurate measurement of the temperature of the cells is another fundamental prerequisite for the effective control of the battery system. The ideal solution would be to directly measure the internal temperature of the cell: in fact, especially for high-capacity cells, a large difference between the internal temperature and the external temperature measured on the cell packaging is expected. During a thermal runaway event, the internal temperature of the cell can rise up to hundreds of degrees Celsius in a very short time interval. On the other hand, the external temperature of the cell packaging can remain essentially unchanged for a time interval that can be too long for early detection of the event [24,25]. Since standard temperature sensors such as thermo-couples and thermo-resistors, even if characterized by relative low cost and sufficient accuracy [26], are difficult to integrate into Li-ion cells to measure the internal temperature, approaches based on the measurement of internal impedance [27,28] and electro-thermal models are emerging [29]. Recently, thanks to the miniaturization and price decrease of printed circuit boards (PCBs), EIS measurement can also be adopted online, with no need of turning the battery pack off. With this method, working in both single- or multiple-frequency acquisition, it is possible to collect information about the aging of the cell through the control of some parameters (SoH, SoC, etc.). The above methods can be used for improving the control strategy implemented in the BMS, but because they require a certain measurement time, they are not suitable to monitor the battery system to prevent thermal runaway events.

On the other hand, the degradation phenomena that can compromise the safety of Li-ion cells are associated with mechanical effects such as the deformation of the cell packaging or gaseous emissions that can anticipate a thermal runaway event. These phenomena have to be monitored using mechanical and chemical sensors integrated in the battery system, but this still remains a difficult task to be realized at an industrial level. An even more advanced topic is related to the in-situ chemical sensors installed directly in the cell to monitor in real time its safety using the detection of specific chemical markers indicating the occurrence of degradation processes on the electrode or electrolyte materials. This would improve the safety of automotive battery systems in a dramatic way, eliminating the risk of sudden and unpredictable thermal runaway events. This research field is very active and is evolving very rapidly. Wei et al. recently published an extensive review devoted to future management systems and sensors for smart batteries [20]; on the other hand, their analysis does not include the chemical sensors and the optical techniques for estimating the mechanical deformation of Li-ion cells. For the above-mentioned reasons, the present paper reviews the most common mechanical and chemical sensor technologies suitable to be integrated in automotive battery packages for real-time SoH and safety monitoring.

## 2. Mechanical Sensors

Li intercalation and deintercalation phenomena occur in the crystal structures of the anode and cathode during the electrical cycling of a Li-ion cell. In particular, a tensile stress was observed when lithium intercalated into the anode material. On the contrary, a compressive stress occurs during the reverse process [30]. Moreover, state-of-the-art high-capacity anodes for Li-ion cells are mainly based on silicon/graphite composites and, since a huge volume change is associated with the lithium alloying with silicon [31,32], stress is placed on the electrodes and dimensional changes of the cells. In addition, the changes of crystal structure and lattice parameters associated with the intercalation of lithium in cathode materials can produce size variations during the charge and discharge processes [30]. Finally, collateral reactions to the normal intercalation and deintercalation of lithium in electrodes (Solid Electrolyte Interphase formation, lithium plating, etc.) produce the formation of new species (gases, solid layers etc.) and may determine a pressure increase inside the cell packaging with consequent dimensional variations of the cell packaging. Both the formation of gaseous species and the crystal structure variation of the cell materials induce stresses on the cell. For this reason, the monitoring of the dimensional variations of the Li-ion cells during cycling is important for the determination of the SoH and for the prevention of safety issues. State-of-the-art diagnostic systems for the in-situ measurement of strain are based on interferometry, piezoelectric effect, laser beam reflection, atomic force microscopy (AFM), and scanning tunnelling microscopy (STM) [33].

### 2.1. Optical Techniques

Three main optical techniques were used in the literature for the detection of in-operando strain in Li-ion cells:

- Digital image correlation (DIC);
- Laser beam position detector (LBPD);
- Multi-beam optical stress sensor (MOSS).

**Figure 3:**
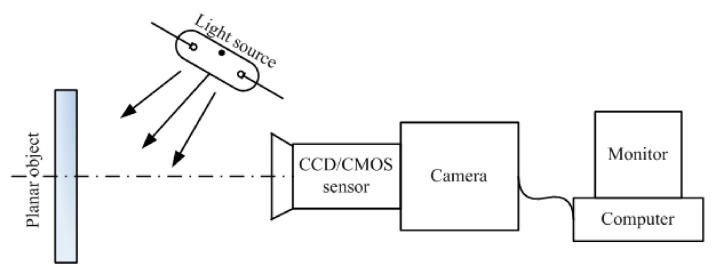
Due to its simple experimental setup, 2D-DIC is ideal to measure the in-plane deformation of an object. The technique is based on the illumination of the object under test using a light source: the image of the surface of the object is captured by a digital camera: the comparison of the undeformed and deformed surface images through numerical digitization software allows for the precise measurement of the in-plane deformation. High-quality cameras are required to obtain accurate measurement of strain. Figure reproduced from [38].

The DIC technique is based on the acquisition of images with a camera sensor: a series of images is recorded at a regular time step, in order to keep track of the dimensional variations of the object under inspection. Using an image processing software, the distribution of dimensional changes of the Li-ion battery at different states of charge is obtained. The resolution of DIC is in the order of μm, depending on the resolution of the camera [34]. The operating principle of DIC is sketched in Figure 3. Historically, DIC was mainly used to characterize the strain in Li-ion cells electrodes [35,36,37].

**Figure 4:**
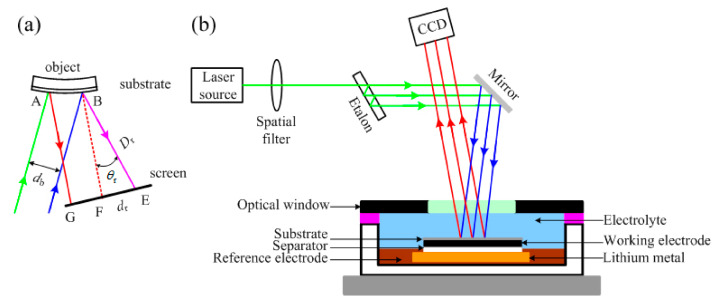
Theory of MOSS technique. (**a**) If two light beams strike on two different positions of a curved object under measurement, the distance between their reflections depends on the curvature of the reflecting surface. The curvature radius R${}_{k}$ is linked to the distance between the reflections d${}_{r}$ according to R${}_{k}$ = 2D${}_{r}$d${}_{b}$/(rcos$\theta_{r}$). (**b**) Using more than two parallel light beams, the distance between adjacent beams can be mediated to reduce the measurement error. A linear array of multi-beams can be obtained using an etalon, an optical element having highly reflective and parallel faces. Using this approach, it is possible to monitor the evolution of stress on a planar or curved objects. Figure reproduced from [38].

LBPD and MOSS are optical techniques useful for performing curvature measurements useful to study the bending of the surface of Li-ion cell electrodes. MOSS is the most recent and applied method and is less affected from vibrations. The operating principle of MOSS is reported in Figure 4.

Sethuraman et al. [31] applied MOSS to study dimensional changes associated with electrical cycling of Li-ion batteries. They performed in-situ measurements of bi-axial Young modulus of an Si thin-film electrode as a function of Li concentration. During intercalation/deintercalation, an elastic stress was generated as a function of the SOC. Optical techniques have several advantages and drawbacks: DIC is fast and inexpensive, and LBPD and MOSS are characterized by an easy data processing and allow the measurement of curvature. The main disadvantage of all the above-mentioned techniques is that they can be used, thanks to special setups, only to characterize electrodes or single cells, so the integration in the battery management systems (BMS) of commercial battery systems is not possible.

### 2.2. Strain Gauges

**Figure 5:**
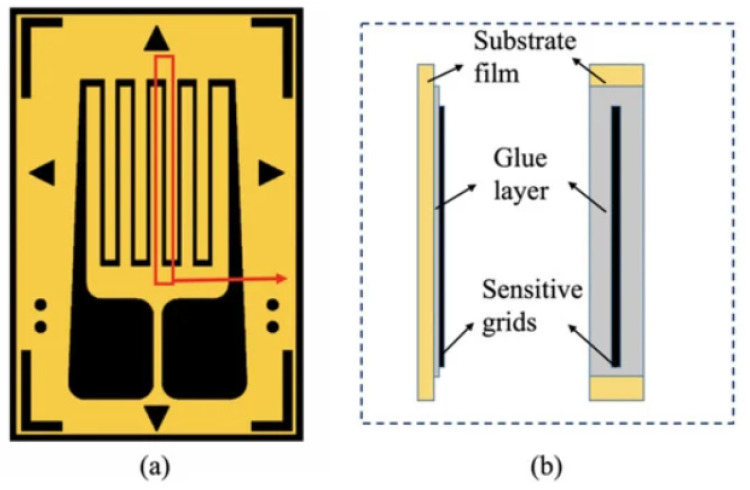
Front (**a**) and side (**b**) views of the typical structure of a resistive metallic strain gauge. Figure reproduced from [40].

Strain gauges are low-cost resistive sensors used to measure the strain of an object. The most common setup consists of a metallic pattern deposited on an insulating flexible support. The sensor is attached to the object under investigation using a strong adhesive. Figure 5 reports an example of resistive strain gauge. If the object suffers a deformation, the electrical resistance measured along the pattern changes. According to [39], the resistance change can be properly put in relation to the strain using a proportional factor known as gauge factor *GF*:

(1) $$GF=\frac{\Delta R/R}{\Delta L/L}=\frac{\Delta R/R}{\epsilon }$$

where *R* is the Ohmic resistance, *L* the length, and ϵ the strain.

**Figure 6:**
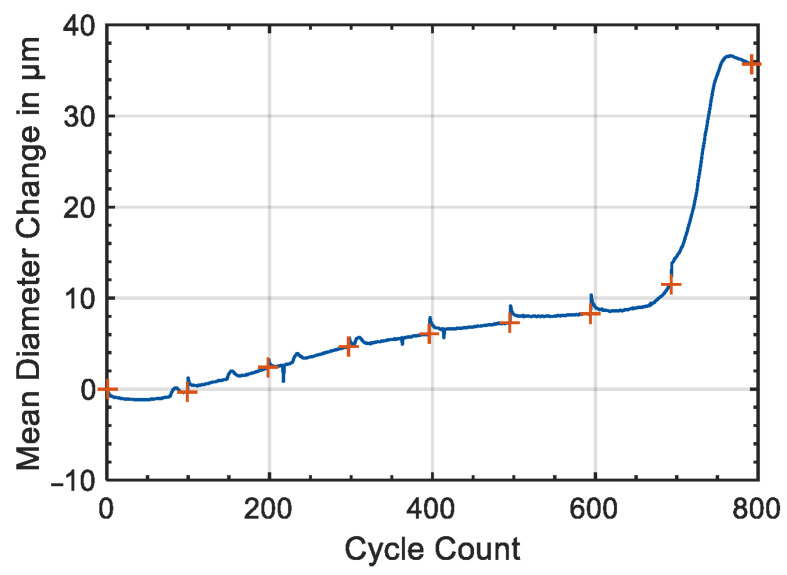
Mean diameter change measured with a strain gauge as a function of cycle count. Orange crosses highlight the time of the check-up. Figure reproduced from [41].

The sensitivity of a strain gauge is related to the relative variation of resistance associated with the strain and depends on the material used to build the conductive pattern. The most common and cheapest strain sensors are based on metals alloys such as Cu-Ni and Ni-Cr, showing a gauge factor ranging between 2 and 5. More recent sensor concepts include the use of wide band gap semiconductors (such as SiC and GaN) and carbon nanotubes, showing piezoresistive and piezoelectric responses [40]. Those materials allow for the reaching of gauge factors and detection limits several order of magnitudes lower than metals. Numerous applications of strain gauges for the monitoring of dimensional variations induced by cycling in Li-ion cells are reported in the literature. Figgemeier et al. applied a strain gauge to the external case of a cylindrical cell with the standard 18,650 format [41]. They concluded that the diameter change has two components, irreversible and reversible, both increasing with the cell ageing. The mean diameter change as a function of the cycle number is reported in Figure 6. The dimensional changes can be understood in relation to typical ageing mechanisms such as increase of the thickness of the anode due to the growth of SEI and the deformation of the jelly-roll of the cell packaging due to the internal stress. The authors found a direct correlation between the diameter change and the capacity loss of the cell and suggested the use of strain gauges as diagnostic tools for predicting sudden cell failure and possible safety problems.

**Figure 7:**
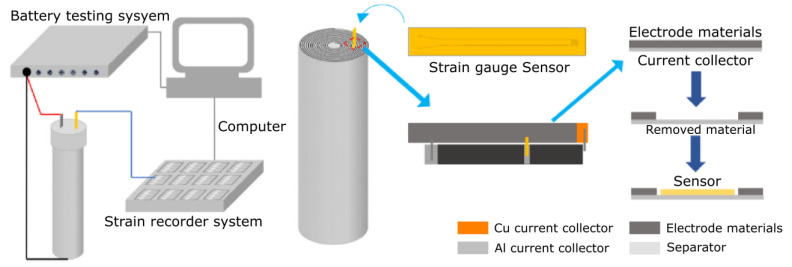
Integration of thin film strain gauge sensor in current collector of the positive electrode of the cell jelly roll. Reprinted with permission from ref. [42]. Copyright 2022 Elsevier Ltd.

More recently, Wang et al. [42] developed a new concept of strain gauge compatible with the in-situ integration in cylindrical 18,650 cells. Figure 7 shows the approach used for the integration of the sensor in cathode layer of the cell: to create the space for placing the thin film strain gauge, a small area of the active material of the cathode was removed from the aluminum support using N-Methyl pyrrolidone (NMP). The sensor was attached to the middle of the width direction in the aluminium current collector and was located in the middle of the radial direction of the jelly roll of the cell.

**Figure 8:**
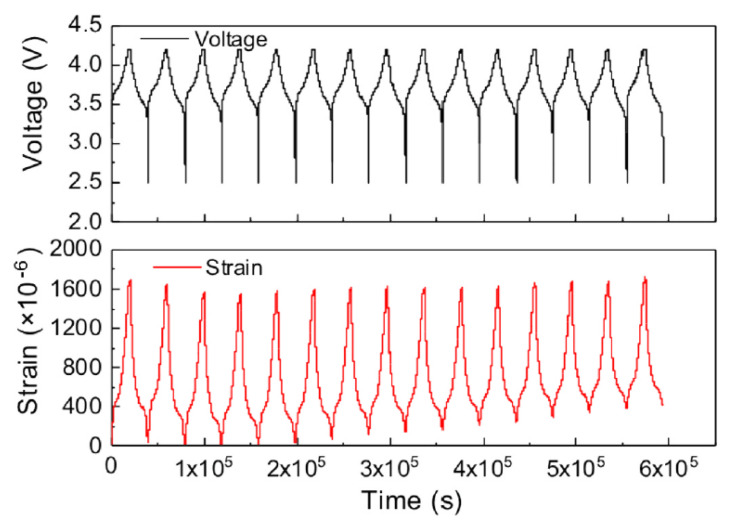
The cycle performance of the instrumented Li-ion cell and the evolution in-operando circumferential internal strain during the first 15 cycles. It is evident that a certain part of the strain is not recovered after the discharge. Reprinted with permission from ref. [42]. Copyright 2022 Elsevier Ltd.

Using the integrated strain gauge, the authors were able to correlate the internal strain of the cell with the SoC (see Figure 8) and to the charge/discharge cycles sustained by the cell. They proposed this approach as a tool to monitor the strain and safety of Li-ion cells in realistic operating conditions since a comparison of the performances between a regular cell and the one with the sensor integrated shows no significative performance differences. Thanks to their low cost [43] and to their constructive simplicity and robustness, strain gauges represent an enabling technology for the monitoring and the development of fault prediction models for automotive battery systems. On the other, even if the current unit cost of suitable components ranges between 1 and 3€ (obtained by direct quotations), the integration of SG in each cell of an automotive battery package remains impractical.

### 2.3. Fiber-Optic Sensors

**Figure 9:**
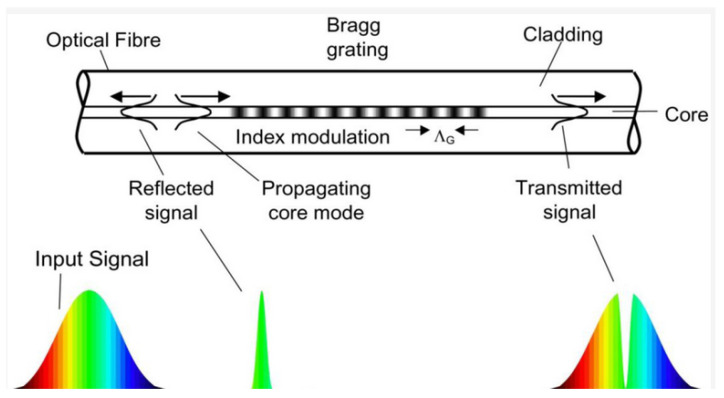
The fiber Bragg grating (FBG) is a wavelength-dependent light filter obtained by creating a periodic refractive index grating, with spacing of the order of a wavelength of incident light, within the core of an optical fiber. If monochromatic light is sent through the grating, the total reflection condition is satisfied when $\lambda_{B}=2n\Lambda$, where *n* is the effective refractive index of the grating, $\lambda_{B}$ is the Bragg wavelength, and Λ is the period of the grating. If a polychromatic light beam is sent to the grating, a reflected spectrum whose centre wavelength is $\lambda_{B}$ is reflected and the remaining portion of light is transmitted. Since the grating period is dependent on the operative temperature and on the deformation of the fiber, the measurement of the shift of Bragg wavelength can be used to sense strain and temperature with a great accuracy. Reproduced from [51].

Fiber optic (FO) sensors are very promising for the monitoring of battery packages in automotive applications. Optical fibers have many advantages with respect to conventional electric cabling: they are electrically insulating and immune to electromagnetic noise; if compared to standard copper cables, they are much lighter; and finally, they are are resistant to corrosion and, if made of polymeric materials, they can resist aggressive chemical species such as hydrofluoric acid, one of the most common products of the decomposition of the cell electrolyte during thermal runaway events [44]. Moreover, the reduced thickness enables the monitoring of the individual cells of the battery package without adding significant size and weight to the system. Optical fibers can be coupled to a wide range of sensors, allowing for the measurement of strain and temperature [45,46,47] acoustic emission and formation of chemical species that can reveal the initiation of degradation processes [48,49]. Fiber optic Bragg grating (FGB) sensors represent the most interesting technology for integration in automotive applications [45]. An FBG sensor is a short segment of optical fiber that reflects selected wavelengths of light and transmits all others (see Figure 9 for details). The sensor is made by inducing a periodic variation in the refractive index of the fiber core, which generates a wavelength-selective dielectric mirror. In case of deformation of the sensor, the interference of light in the fiber is modified, and this effect can be used to measure the strain induced by cycling in the cell electrodes. Another possible application is the fast measurement of temperature with sensitivity in the range of 0.1 °C [50]. On the other hand, this characteristic can be also a drawback since a careful decoupling of the response of the sensor to mechanical strain and temperature is required. A detailed cost analysis for the application of FBG sensors in automotive applications was reported in [49], where a cost of $10 for each FBG sensor and $3 for each meter of optic fiber were estimated. Moreover, the treatment of the optical signals requires the use of dedicate acquisition hardware coupled to the BMS. For the above reasons, considering that an automotive battery package can contain up to 400 cells (or thousands of low-capacity cells in applications like Tesla vehicles), the cost of monitoring each cell of the package remains prohibitive.

## 3. Chemical Sensors

The dimensional variation of the cell is not the only indicator that can be used to evaluate the state of health of Li-ion cells. In fact, the formation of gaseous chemical species that form inside the cell and vent outside is another important phenomenon to be taken into account. This is especially true before and during thermal runaway events. Thermal runaway implicates several sequential processes: SEI decomposition, anode collapse, reaction of intercalated lithium with the electrolyte, separator melting, decomposition of the cathode active material and of the lithium salt of the electrolyte, electrolyte solvent oxidation, reaction of the intercalated lithium with the binder, and collapse of the ceramic coating of separator. This chain of events determines a violent explosion or firing of the cell. During the development of these chain reactions, peaks of 120 °C can be registered. In these conditions, the compounds present in the SEI will decompose to flammable hydrocarbons, CO${}_{2}$ and O${}_{2}$. The various reactions can be worsened by the presence of O${}_{2}$; furthermore, oxygen is a combustion adjuvant, another threat for lithium-ion batteries safety. Several decomposition reactions can occur inside a cell: ROCO${}_{2}$Li and (CH${}_{2}$OCO${}_{2}$Li)${}_{2}$ can be taken as examples of two of the main components of SEI [52]:


ROCO_2_Li → CO_2_↑ + ROLi

$${\left({\mathrm{CH}}_{2}{\mathrm{OCO}}_{2}\mathrm{Li}\right)}_{2}\to {\mathrm{Li}}_{2}{\mathrm{CO}}_{3}+{\mathrm{CO}}_{2}↑+\frac{1}{2}O_{2}↑+C_{2}H_{4}↑$$

Once lithium is intercalated into the anode’s structure, it can come in contact with the electrolyte after the anode collapses; this can stimulate an exothermic reaction producing some flammable hydrocarbon. Common solvents used in lithium-ion batteries are ethylene carbonate (EC), dimethyl carbonate (DMC), propylene carbonate (PC), and diethyl carbonate (DEC) [53]:

C_3_H_4_O_3_(EC) + 2Li → Li_2_CO_3_ + C_2_H_4_↑

C_3_H_6_O_3_(DMC) + 2Li → Li_2_CO_3_ + C_2_H_6_↑

C_4_H_6_O_3_(PC) + 2Li → Li_2_CO_3_ + C_3_H_6_↑

C_5_H_10_O_3_(DEC) + 2Li → Li_2_CO_3_ + C_2_H_6_↑ + C_2_H_4_↑

As a consequence of the increase of electrochemical side reactions ratio, the separator melts, the decomposition of the active material of the cathode occurs, and the lithium salts that compose the electrolyte start to decompose to generate toxic and flammable gases. In a common lithium-ion battery composed of lithium-manganese-oxides and LiPF_6_ as the electrolyte, decomposition reactions are typically [54]:


$${\mathrm{Li}}_{x}{\mathrm{Mn}}_{2}O_{4}\to \frac{x}{3}{\mathrm{Mn}}_{3}O_{4}+(1-x){\mathrm{Mn}}_{2}O_{3}+{\mathrm{xLiMnO}}_{2}+\frac{3-x}{6}O_{2}↑$$

LiPF_6_ ⇌ PF_5_ + LiF

Afterwards, the oxidation of organic electrolyte solvent begins. Large amounts of oxygen are produced by reactions that decompose the SEI and other side reactions at the cathode; this can lead to a quick increase of heat generation, as much as the release of a substantial amount of gaseous species and vapors. As mentioned before, common organic electrolyte solvent EC and DEC can undergo these reactions:

4C_3_H_4_O_3_(EC) + 7O_2_ → 6CO_2_↑ + 6CO↑ + 8H_2_O↑

4C_3_H_6_O_3_(DEC) + 9O_2_ → 6CO_2_↑ + 6CO↑ + 12H_2_O↑

The decomposition product of LiPF_6_, PF_5_, can react with vapor to form hydrogen fluoride and phosphorus oxyfluoride, which are harmful and toxic. Furthermore, PF_5_ can react with Li_2_CO_3_, a stable substance composing the SEI, to generate greenhouse gases. Additionally, HF can easily react with Li^+^ present in the electrolyte in order to form LiF and hydrogen radicals [55]. Taking in account the binder, polyvinylidene fluoride (PVDF), which is widely used in lithium-ion batteries and explosive and flammable gases, can be generated by the reaction of intercalated lithium metal with the binder:


$${\left[{\mathrm{CH}}_{2}-{\mathrm{CF}}_{2}\right]}_{n}+\mathrm{Li}\to \mathrm{LiF}+{\left[{\mathrm{CH}}_{2}={\mathrm{CF}}_{2}\right]}_{n}+\frac{1}{2}H_{2}↑$$

Finally, the flammable gases generated and the electrolyte solvent vapor can accumulate causing the rupture of the battery case.

According to Cai et al. [56], the degradation product ratio is highly variable depending on the chemistry and structure of the lithium-ion battery and the abuse conditions. For example, in the case of NMC-based cells at 100% SoC, during overheating, the gas composition varies according to the geometry: in cylindrical cell, the main products are CO_2_ and H_2_; in prismatic cells, CO and Volatile Organic Compounds (VOCs) are the principal degradation products; and in pouch setup, CO and CO_2_ cover more than the 60% of the total products. Considering the thermal abuse of cylindrical NCA-based lithium-ion cells at different SoC values, the product gases are very different: for an SoC between 0 and 25%, the main product is CO_2_, while H_2_ and VOCs are present under 15% and traces of CO are present; with SOC between 50 and 143%, the main product is CO, followed by CO_2_ and H_2_; at 100% SoC, the main product is CO, while H_2_, CO_2_, and VOCs are almost present with the same percentage. During overcharging of cylindrical cells based on the LFP cathode material, the main product is CO_2_ followed by H_2_ and VOCs. Taking these results into account, the main degradation product is CO_2_; for this reason, most of the gas sensors are sensible to this chemical species.

**Figure 10:**
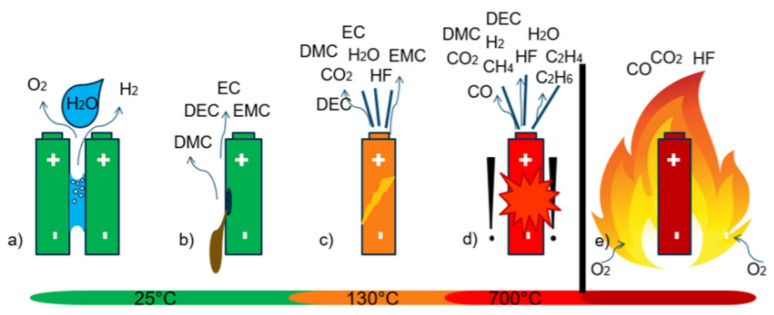
Battery failure modes, which involve the emission of gaseous species: (**a**) electrolysis due to the presence of water between positive and negative poles of the cells, (**b**) evaporation of electrolyte from damaged cells, (**c**) early venting from a failing cell, (**d**) the thermal runway (TR), and (**e**) battery fire. Adapted from [58].

Figure 10 summarizes the main failure events involving the emission of gases from a Li-ion battery. If water is present inside the system, the detection of H${}_{2}$ and O${}_{2}$ also occurs at ambient temperature, due to the electrolysis of H${}_{2}$O. At higher temperatures (from 130 °C), the vaporization of the electrolyte can also lead to venting phenomena, along with electrolyte degradation. In the worst cases, batteries cause thermal runaway (at about 700 °C) with VOCs, CO, and CO${}_{2}$ produced [57,58].

Mateev et al. [59] pointed out that the presence of CO is related to an incomplete combustion of the electrolyte and to the initial temperature rising, while the presence CO${}_{2}$ is associated with intensive oxidative processes. Cell thermal runaway showed that a gas detection method targeting CO${}_{2}$ concentration reacts faster, if compared to temperature monitoring [60]. The detection of both gases can be matched to increase the robustness of the battery fault recognition system. Generally, CO${}_{2}$ sensors include chemical sensors and Nondispersive Infrared sensors (NDIR). Since CO${}_{2}$ and VOCs are major components of the developed gas mixture, it is not necessary to obtain high accuracy from the implemented sensors. Fast detection can be obtained by using low-cost sensors. Today, the literature in this field suggests four main classes of sensors:

- Electrochemical sensors;
- Semiconductor sensors;
- NDIR sensors;
- Chemical sensors:
  - Traditional CO${}_{2}$ and VOC sensors;
  IC-MOF sensors [61] and functionalized double-walled carbon nanotubes [62].

The detection of CO, H${}_{2}$, and VOC is usually performed using electrochemical sensors or metal-oxide semiconductor (MOS) [58]. Both MOS and electrochemical sensors offer good selectivity and excellent accuracy (usually within ±2% of full scale) [63].

**Figure 11:**
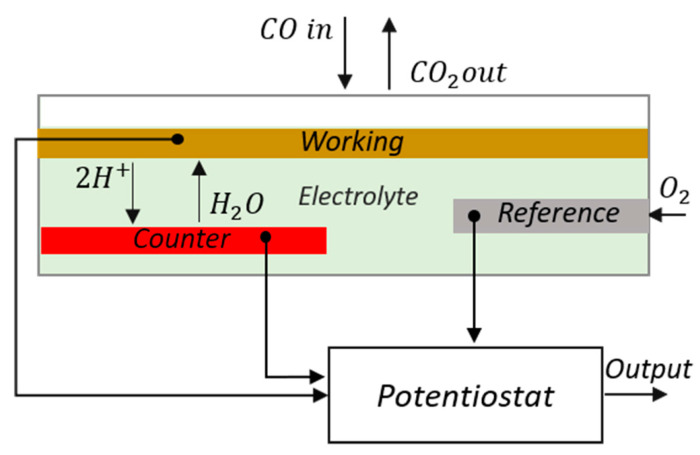
Operating principle of an electrochemical sensor for CO detection. Reproduced from [69].

Electrochemical VOC sensors are amperometric sensors, made of a working (sensing), a counter, and a reference electrode. They require low power and are very compact but suffer from cross-sensitivity to other gases and large signal drift and are affected by low humidity environment. The unit price varies generally between 20 and 30 euros [56]. Conceptually, electrochemical gas sensors are structured as a fuel cell: two catalyzed electrodes are separated by an ionic conductor (see Figure 11). When the gaseous analytes, for example, CO or H${}_{2}$, come in contact with the working electrode, oxidation of the gas will take place, releasing electrons in the electrode and H${}^{+}$ ions in the electrolyte. Connecting the working to the counter electrode using an external circuit, electrons generated at the working electrode will flow through the external wiring to reach the counter electrode. To compensate for this movement of charges, protons generated at the interface between the electrode and the electrolyte will flow toward the counter electrode through the ionic conductor. To complete the redox reaction at the counter electrode, the electrons combine with the protons and the oxygen molecules from the air to form water. Electrochemical sensors work normally in amperometric conditions: a constant external potential is applied between the electrodes, and the current flowing in the device is measured. If the sensor is operated in diffusion-limited conditions, the current depends linearly on the concentration of the gaseous analyte.

**Figure 12:**
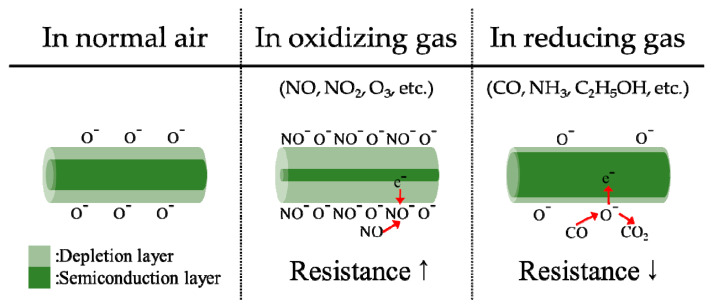
Operating principle of an MOS chemical sensor. N-type metal oxide semiconductors contains oxygen vacancies due to non-stoichiometry. When the sensor is exposed to oxidizing molecules at temperature between 200 and 400 °C, oxygen ions attach on the surface of the oxide, depriving electrons from the conduction band of the material. For this reason, the resistance of the sensing element increases. On the contrary, in presence of reducing molecules, the process is reversed and a decrease of resistivity is observed. Adapted from [70].

Semiconductor sensors work by monitoring the electrical resistance of metal oxide, which is modified by the presence of the gaseous species under testing. The mechanism relies on the surface reactions occurring at high temperature when the gas under analysis comes in contact with a metal-oxide semiconductor. In fact, a change of conductivity of the semiconductor oxide is induced by the absorption of molecule at the surface of the material [64]. The chemi-sorption of oxygen causes an extraction of electrons from the conduction bands of the semiconductor to form surface localized electronic states. This phenomenon determines an increase of the resistivity of the material. On the contrary, if the sensor is exposed to reducing gases, the reaction of surface oxygen species with them or the replacement of the adsorbed oxygen by other molecules can reverse the conduction band depletion and decrease the resistivity. The most common MOS integrate a sensing element made of SnO${}_{2}$ and ZnO [65,66,67]. Literature also reports the existence of other minor species such as Cr${}_{2}$O${}_{3}$ [68], also implemented for the detection of gas species as sensor. Figure 12 illustrates the operating principle of MOS sensors.

Tin dioxide (SnO${}_{2}$) is the most used sensor of this type due to its broad reactivity to VOCs and large changes in resistance. The broad reactivity means that SnO${}_{2}$ semiconductor sensors are sensitive to not only various VOCs but also to NO, NO${}_{2}$, and CO. MOS sensors have a large power consumption and suffer from cross-sensitivity to different gas species and important signal drift (up to 5% per year). The unit price of a semiconductor VOC or CO sensor generally ranges from 5 to 10 euros [56].

**Figure 13:**
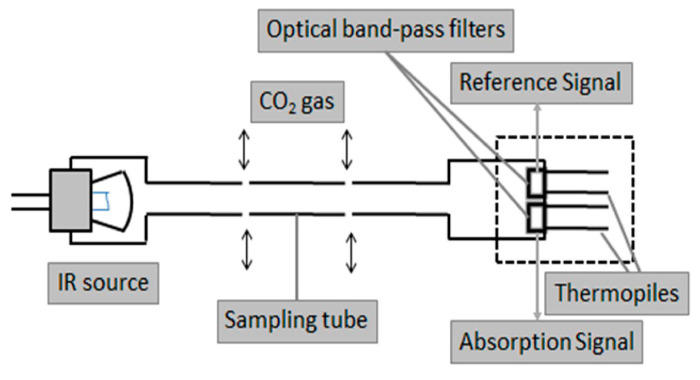
Internal structure of an NDIR CO${}_{2}$ sensor. Constituted by an IR lamp, a perforated pipe in which light interacts with CO${}_{2}$ molecules in the air, and thermopiles provided with optical bandpass windows. Adapted from [73].

Among various possible measurement principles to detect CO${}_{2}$, NDIR sensors show the best compromise in terms of cost (between 8 and 20 euros [56]) and lifetime [71]. In general, the accuracy of NDIR sensors is very good and within 1% of full scale [72]. Moreover, being based on the measurement of wavelengths of the CO${}_{2}$ vibrational spectrum, this sensor technology is very selective and suffers from a very limited signal drift (about 0.15% per year) [56]. See Figure 13 for the layout of a typical NDIR sensor.

Chemical sensors work by using sensitive layers targeted at CO${}_{2}$, with low energy consumption. In this field, quartz microbalance transducers are among the most widespread [74]. Here, a mass variation per unit area is measured by the change in frequency of a quartz crystal resonator. The sensor price is generally from 15 to 35 euros [56]. This class of sensors drift over time with fast degradation. Recently new classes of chemical sensors were suggested by the literature, the IC-MOF (Ionically Conductive Metal-Organic Frameworks) [61] and functionalized double-walled carbon nanotubes [62]. IC-MOF sensor detects Li-ion battery electrolyte leakage. A very fast detection of dimethyl carbonate (50 ppb) and electrolyte (20 nL) leakage can be found within few seconds, where usually Copper ions act as charge carrier.

Another new class of chemical sensors, recently introduced into the literature [62], open up an interesting discussion on the usage of double-walled carbon nanotubes (DWCNTs). If functionalized with 3,5-dihydroxydiazobenzene salt, they showed an interaction for DMC vapor via a hydrogen bond, with significant changes in the conductivity.

## 4. Conclusions

Automotive Li-ion battery packages require the integration of Li-ion cells, a battery management system, and a complex network of sensors. The systems must comply with a demanding list of requirements and have to guarantee, in addition to high energy and power densities, a long cycle life and intrinsic safety. Not all the technologies explored in this review are mature enough to be fully integrated in an automotive battery package. Some technologies could potentially be suitable for large-scale on-board large application, but the current cost or the difficult integration represent the main limitation.

Table 1 compares the main characteristics of commercially available mechanical and chemical sensors characterized by a sufficient technological maturity to be integrated in automotive battery systems. Optical techniques for dimensional analysis are excluded from the table not on the basis of the maturity but because of the difficult integration in a real battery package.

According to our analysis, the available commercial sensors allow for the accurate in-situ measurement of mechanical deformation and composition of vented gases. Their integration in the battery system can complement and improve the robustness of current BMS based on the measurement of temperature and of electrical quantities. Chemical sensors can be an effective solution to reveal battery failures by CO/CO${}_{2}$ detection, but further improvements can be obtained by focusing on the leakage of organic volatile compounds from the electrolyte of the cells in order to achieve an earlier detection of potential failures. The main advantage of chemical sensors is that to monitor the battery system it could be sufficient to integrate a limited number of sensors in selected positions (in the limit case just one sensor): this would have a limited impact on the cost and complexity of the battery package. The integration of mechanical sensors in the single cells, even in the case of low-cost strain gauges, still appears prohibitive considering the large number of cells composing the current automotive battery packages (some hundreds in case of high-capacity cells, some thousands for solutions based on small cylindrical cells such as in Tesla vehicles). On the other hand, the application of mechanical sensors for in-operando studies of Li-ion cells is an extremely powerful approach to improve battery modelling tools and associated BMS strategies.

## Figures and Tables

**Table 1:** Comparison of different sensor technologies for mechanical and chemical monitoring of Li-ion battery systems.

Measured Property	Sensor Type	Cost	Advantages	Drawbacks
Deformation	FBG	High	High accuracy, no electro-magnetic interference	Coupled response to temperature and strain
	SG	Low	Low cost, good accuracy, and mature technology	Sensitive to electro-magnetic interference
Gas composition	MOS (CO, VOC)	Low	Compactness, easy integration	Cross sensitivity to different gases, medium signal drift, and high power consumption
	Electrochemical (CO,VOC)	High	Compactness, easy integration	Cross sensitivity to different gases, severe signal drift, and affected by moisture
	NDIR (CO_2_)	Medium	Selectivity, long lifetime, and limited signal drift	Large dimensions if compared to MOS and electrochemical sensors

## Data Availability

Not applicable.

## Abbreviations

The following abbreviations are used in this manuscript:

SoC	State of Charge
SoH	State of Health
EC	Ethylene Carbonate
DEC	Diethyl Carbonate
DMC	Dimethyl Carbonate
PC	Propylene Carbonate
FO	Fiber Optic
BMS	Battery Management System
OCV	Open Circuit Voltage
SEI	Solid Electrolyte Interphase
VOC	Volatile Organic Compounds
MOS	Metal-Oxide Semiconductor
NDIR	Non-Dispersive Infrared
AFM	Atomic Force Microscopy
STM	Scanning Tunnelling Microscopy
DIC	Digital Image Correlation
LBPD	Laser Beam Position Detector
MOSS	Multi-beam Optical Stress Sensor
FBG	Fiber Bragg Grating
NMC	Lithium Nickel Manganese Cobalt Oxide
NCA	Lithium Nickel Cobalt Aluminium Oxide
LFP	Lithium Iron Phosphate Battery
IC-MOF	Ionically Conductive Metal-Organic
DWCNTs	Double-Walled Carbon Nanotubes
